# Collaborative design approach to identify research and innovation needs within the European smart building community

**DOI:** 10.12688/openreseurope.15174.2

**Published:** 2024-10-15

**Authors:** Clémentine COUJARD, Karine Laffont Eloire

**Affiliations:** 1 DOWEL Innovation, Sophia Antipolis, France, 06901, France

**Keywords:** smart buildings, collaborative design, participative design

## Abstract

**Background:**

Upgrading the level of smartness in buildings can significantly contribute to improve our quality and sustainability of living, through increased energy efficiency, optimised resource management, and improved air quality and comfort. However, the fragmented nature of the sector makes it challenging to identify what is technically, socially and economically achieved today in Europe in terms of building smartness, and what should be developed and financially supported tomorrow to accelerate the roll-out of smart and energy efficient buildings.

**Methods:**

This paper introduces the collaborative process developed to involve a large community of experts in detecting and formalising research and innovation gaps related to smart buildings. This process is composed of four successive steps: 1) a communication phase to invite volunteer experts to join the proposed task forces; 2) The organisation and facilitation of online brainstorming workshops to identify research & innovation (R&I) gaps; 3) the collective drafting of a white paper synthesising the brainstorming outcomes; and 4) an open consultation to collect additional external feedback before finalising the white paper.

**Results:**

The collaborative process developed was tested over 18 months and implemented on 12 different topics relying on 27 brainstorming workshops. Building on the collective knowledge of 135 participants, it enabled identification a significant series of R&I gaps related to smart buildings.

**Conclusions:**

The collective sessions as well as the open consultation phases showed overall some clear convergence on the gaps identified. It can therefore be concluded the outcome of the collaborative process reached a consensus among the targeted innovation community. The feedback collected on the process, shows that the frequency, duration and attendance of the brainstorming workshops proposed were very relevant, while the selection of online participatory tools could still be improved. This process could be replicated in other frameworks where research and innovation gaps are sought for.

## Plain language summary

The deployment of smart building solutions throughout Europe faces some barriers and challenges, and the related innovation efforts need to be supported by the public authorities.

This paper describes a collaborative design process implemented in 2021–2022 to involve the European smart building community in identifying what type of research and innovation activities should be funded in the next years.

## Introduction

The built environment impacts our lives in many ways: it influences our living, occupational or mobility behaviours, but also triggers significant environmental impacts, which in turn affect our health and wellbeing ( Altomonte *et al.*, 2020).

Upgrading the level of smartness in existing and future buildings can significantly contribute to improve our quality of lives and sustainability of consumption behaviours, through increased energy efficiency, optimised resource management, improved air quality and comfort ( Hoy *et al.*, 2016).

Numerous developments are underway to upgrade the intelligence of buildings ^1^, from new technological solutions relying on information and communication technology (ICT), sensing and automation, to performance assessment schemes such as the Smart Readiness Indicator. However, those ongoing efforts are very dispersed along the European construction value chain, due to the sector’s fragmented structure, and diversity of trades involved in implementing and operating smart solutions in buildings ^2^. This context makes it challenging to get an overview of what is achieved today in Europe, and what should be developed and financially supported tomorrow to accelerate the roll-out of smart and energy efficient buildings.

The SmartBuilt4EU project aims to consolidate the European innovation community in smart buildings in view of sharing good practices and formalising recommendations for further research and innovation. This paper introduces the collaborative process designed and implemented to involve a large community of experts in the elaboration of White Papers identifying research and innovation gaps related to smart buildings.

### Definition of smart building

Building’s smartness refers to the ability of a building or its systems to sense, interpret, communicate and actively respond in an efficient manner to changing conditions in relation the operation of technical building systems or the external environment and to demands from building occupants (Al Dakheel *et al.*, 2020). The potential of smart technologies in the building sector was heavily emphasised in the 2018 revision of the European Energy Performance of Buildings Directive (EPBD).

### The SmartBuilt4EU project

The SmartBuilt4EU project is an EC-funded project led by ECTP, the European Construction, built environment and energy efficient building Technology Platform. SmartBuilt4EU aims to foster collaboration between stakeholders of the smart building ecosystem, promote their innovations, and identify research and development (R&D) gaps to support the further uptake of smart buildings. Two major outputs of the project are a strategic research and innovation agenda (SRIA) and a set of policy recommendations to foster the further deployment of smart solutions in the built environment. Those documents, to be published in March 2023, are elaborated based on a collaborative process enabling to gather inputs from diverse members of the smart building innovation community (SBIC), detailed hereafter.

## Research method

### Objective and overall structure of the collaborative design process

The objective of the collaborative design process developed within SmartBuilt4EU was to gather and formalise inputs from the smart building innovation community on the research and innovation needs that should be supported and financed by European public authorities in the coming years.

The needs considered were organised in two categories in view of structuring the discussions with the community: “R&D gaps” that include research, development, and demonstration activities; and “go-to-market gaps” covering standardisation, certification, industrialisation and upskilling needs, as well as regulatory evolutions.

The participatory approach developed relies on two key components: the setup of four voluntary task forces, and a harmonised workflow for each task force to elaborate successive white papers during a limited time span (six months). Setting up four task forces enables to address four different dimensions of the smart buildings’ domain and gather participants around specific topics matching their own interest and expertise. The elaboration of white papers enables the authors to formalise the brainstorming outputs into a format that is directly exploitable for the construction of the final SRIA. Framing each white paper’s work into a six-month period enables us to keep participants involved and motivated towards a short-term goal. The next sections introduce the four task forces and the different steps towards the white papers’ elaboration.

### The four task forces

The participation to task forces is open to any stakeholder and on a voluntary basis. The four task forces respectively address distinct but complementary themes related to smart buildings. Their objective is to investigate three specific topics and produce a related white paper highlighting research and innovation gaps (R&I gaps). This is performed in a sequential manner along three semesters. The respective themes of task forces were defined as follows:

- Task Force 1 addresses the interactions between the smart building and its users (occupants, operators): it covers the issues of end-user acceptance and attractiveness of smart solutions; enhanced quality of life for occupants; and operational feedback to the end-user towards behavioural change.
- Task force 2 addresses the optimal integration and use of smart solutions to allow an efficient building operation: it covers interoperability; optimisation of building costs; and smartness to reduce building’s environmental impacts.
- Task force 3 addresses the interactions between the smart building and its external environment: it covers the provision of power flexibility to the grid, new energy practices and communities, and data-driven indicators for smart buildings.
- Task Force 4 addresses crosscutting issues, with specific focus on business models and financing; data governance and cybersecurity; and education & upskilling.

Those themes were designed to cover a wide range of aspects related to smart buildings, from social and societal dimensions to technical and technological questions as well as business and financial ones ^3^.

Each six-month topic is co-chaired by two members of the community: one from the SmartBuilt4EU consortium, and the other from the expert board that was set up at the project start ^4^. The co-chairs are involved in the definition of the task force topic, the preparation and facilitation of the brainstorming workshops, and drafting of the white paper. A ‘task force secretariat’ is ensured by DOWEL Innovation to plan, organise and facilitate the overall workflow.

### The collaborative workflow

The collaborative process implemented by each task force relies on four main components:

1. communicate on the launch of a topic and the possibility to join the task forces, so as to gather a maximum of stakeholders
2. organise and facilitate three online brainstorming workshops to progress towards the identification of R&I gaps
3. collectively draft a white paper, which then undergoes an internal review process
4. conduct an open consultation to collect additional external feedback before finalising the white paper

Step 1 on communication exploits all dissemination channels enabled by the SmartBuilt4EU project (emailing to the community, social media posts, news on website, promotion during conferences and events).

Step 2 is the critical phase to collect valuable material for the white papers. The online workshops are organised using a visio-conference tool (Microsoft Teams) and an online brainstorming tool ( ConceptBoard ^5^) (a free open-source alternatives with similar features can be found at OPENBOARD) enabling to collect written contributions from all participants. The three successive workshops follow a progressive approach towards the identification of R&I gaps:

- workshop 1 focuses on scoping the exact topic investigated and discussing state of the art
- workshop 2 investigates the barriers and drivers related to the defined topic
- based on this analysis, workshop 3 focuses on identifying the related R&I gaps.

Each workshop has a duration of approximately 90 minutes. To structure the discussions and outputs, the brainstorming sessions are broken down into short successive time slots. For instance, barriers and drivers are categorised into ‘value chain’, ‘social’, ‘technical’, ‘economic and financial’, and ‘legal and regulatory’: a dedicated 5–10 minute brainstorming timeslot is allocated to each category.

In an equivalent process, the brainstorming about R&I gaps is broken down into ‘R&D’, ‘demonstration’, ‘scaling up and industrialisation’, ‘standardisation/certification’, and ‘regulation’ categories, with successive timeslots. Once all categories are addressed, the facilitator proposes to cluster the different inputs provided into key ideas, that are submitted to a vote. This enables to highlight which gaps are considered as priorities by participants.

**Figure 1.**
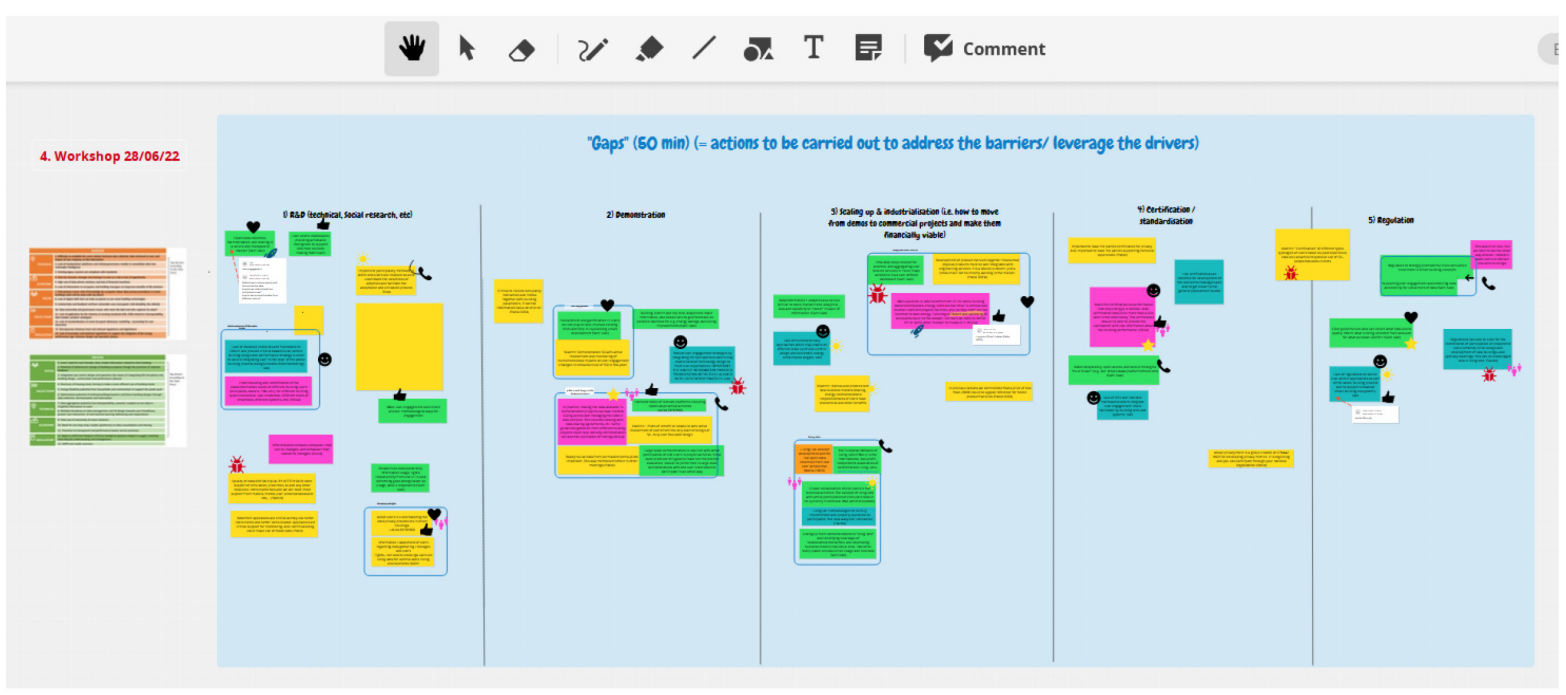
Screenshot of a ConceptBoard page to facilitate the session on identifying and prioritising R&I gaps on topic 1A.

The ConceptBoard, the online participatory tool to collect written contributions from participants, was structured according to the categories listed above. Figure 1 shows a concept board used to identify research & innovation gaps for topic 1A. The orange and green tables on the left side ( Figure 1) are respective reminders of the barriers and drivers identified during the previous brainstorming. The five columns in the blue frame reflect the 5 categories of R&I gaps listed above. The colour notes are the inputs from participants. The blue rectangles cluster some inputs into common ideas. The small icons (thumb, heart, etc) represent the vote of participants of the ideas proposed.

In step 3, the secretariat of each task force initiates a draft white paper formalising the brainstorming outputs. The document is then shared with task force members to collect additional feedback and inputs, in particular regarding lessons learnt from EU-funded projects. An internal review of all four white papers is finally performed by project partner VITO to check the coherence between white papers.

In step 4, the draft white papers are released online and disseminated, together with an online questionnaire to collect feedback from stakeholders not directly involved in the task forces. Their feedback is then processed towards the finalisation of white papers.

### Additional knowledge sharing through webinars and workshops

In addition to the collaborative workflow described above, a total of 33 EU-funded projects were invited to present their results, lessons learnt and good practices through webinars and workshops. Three webinars were co-hosted with Build Up from February 2021 to April 2022, one workshop was organised within the Sustainable Places 2022 conference in September 2022. These presentations, which were structured to encourage the exchange of real-life feedback from EU-funded projects, provided valuable inputs to the “Lessons learnt” sections in the white papers.

## Results and analysis

The collaborative process developed by SmartBuilt4EU started at the end of February 2021 with an online workshop officially launching the task forces and attended by close to 50 participants from both industry and research. The first round of white papers was elaborated from February to September 2021, the second round from September 2021 to March 2022, and the third and final one from April to October 2022.

### Participants

In total, 135 persons contributed to the twelve white papers produced, covering 23 European countries. Task force members featured a diversity of profiles: 45% come from public and private research organisations, 39% from business companies, 13% represent associations (European sector associations, national or regional associations or clusters) and 3% are certification or standardisation bodies. Many of them are involved in EU-funded projects (31 participating projects were referenced).

An analysis of the participants of topic A of task force 1 (topic ‘End user acceptance and attractiveness’) showed that 64% were male and 36% female.

With regard to the brainstorming workshops, the average level of participation was of 21 persons. For each topic, the first workshop (out of three) attracted the most people (28 in average), which can be explained by the fact that these ‘topic launch’ workshops were better promoted than the following ones. The average participations in workshops 2 and 3 were respectively 19 and 18 attendees, showing a persistent interest from members. Participation was also relatively well spread between task forces, with averages ranging from 13 to 19 participants.

Considering the open consultation process (step 4) very low feedback was observed, despite the significant communication actions undertaken. Seven answers were obtained on the first batch of white papers (topic A), two on the second batch and five on the third batch. Though it is difficult to explain the reasons of such low feedback, it can at least be concluded that engaging people was more efficient through online workshops than through an online questionnaire, no matter how brief and concise it was.

### Topics covered and white papers produced

Table 1 lists the twelve topics investigated by the task forces. Topics A were the first round of topics, topics C the last round.

**Table 1.** Topics covered by the task forces.

	Task Force 1: Interactions with users	Task Force 2: Efficient building operation	Task Force 3: Interactions with the external environment	Task Force 4: Crosscutting issues
Topic A	End user acceptance and attractiveness	Interoperability	Providing flexibility to power grids	Financing and business models
Topic B	Occupant-centric building for improved quality of life	Optimised building costs	Smart building as enabler of new energy practices and communities	Data governance, security and privacy
Topic C	Responsive end-user	Smartness to reduce building’s environmental impacts	Data-driven indicators for smart buildings	Education and upskilling

**Figure 2.**
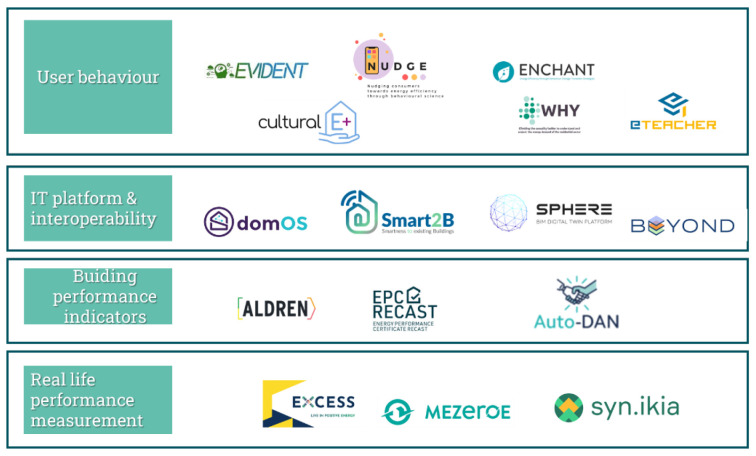
State of the art: mapping performed by the white papers authors about the identified EU-funded projects related to topic 1C: responsive end user.

The structure of each white paper produced reflects the sequence of brainstorming workshops: 1) scope of the paper and state of the art; 2) barriers and drivers, 3) R&I gaps. Figure 2, Table 2 and Table 3 gives an abstract view on how those elements were synthetised in a visual format suitable for dissemination, with the example of topic 1C (‘responsive end user’).

**Table 2.** Synthesis of the barriers identified with task force members during an online brainstorming session for topic 1C: responsive end user.

Category	Barriers
Technical	Difficulty to establish the exact relation between data collected, data retrieved to user, and impact of user response to this information
	Lack of mechanisms/ platforms and related governance models to consolidate data into actionable intelligence
	Existing legacy systems not compliant with standards
Economic	Data has become strategic and sharing it is seen as a risk or loss of opportunity
	High cost of data-driven solutions and lock of financial incentives
Social	Data privacy issues: lack of knowledge by occupants about data privacy procedures in smart buildings (who will do what with my data?)
	Lack of digital skills that can help occupants to use smart building technologies
	Limited data and feedback on/from vulnerable users (occupants with disability, the elderly)
Value chain	Data ownership and governance issues: who owns the data and capture its value?
	Lack of application by the industry of existing standards (ISO, CEN) related to interoperability, data models, product catalogues
	Lack of standardisation of Occupant Behaviour modelling – accounting for user diversities
Regulation	Discrepancies between local and national regulations and legislations
	Lack of normative and technical regulations to support the mitigation of the energy performance gap between design and operation phases

**Table 3.** Synthesis of the R&D gaps identified with task force members during an online brainstorming session for topic 1C: responsive end user.

Category	Research and development gaps
Research and development	Implement participatory methods involving end-users, to: - Understand the conditions of adoption of smart solutions - Understand and identify the data/information needs of different building users (occumpants, owners, facility managers, etc), for different building types (residential/ tertiary, with different levels of smartness) - Identify occupants’ behaviours that can be modified and those that cannot - Design solutions providing pertinent and actionable information to users in line with their energy practices
	Clarify data ownership, usage and rights when data is aggregated and user in integrated services (platforms)
	Define how to share openly and harmonise the data: how to collect data from multiple sources? How to harmonise the data from different nature?
	Apply artificial intelligence, modelling, to help fill the quality gap on real data collected
Demonstration	Deploy living labs (permanent pilots) giving open access to real data on building’s monitoring and user perspectives
	Implement large scale demonstrators with emphasis on the active participation of end users, from the design phase. This implies engagement strategies relying on social science and humanities, and the use of techniques such as gamification
	Systematically integration in demonstration the monitoring of occupancy and weather data together with building parameters to ensures data quality

### Feedback from participants on the participatory process implemented

On June 4th 2021, after the first series of workshops was conducted on topics A, an online questionnaire ( Coujard, 2022b) was proposed to participants during the last online session of task force 1 to collect feedback on the collaborative process implemented. The questionnaire was designed so that 1) it could be filled-in ‘live’ in less than five minutes by participants; 2) participants would evaluate some specific aspects of the proposed workflow through multiple choice questions (about the frequency, duration and audience of the sessions, and the tools used); and 3) participants could add a free comment on how to improve the collaborative process.

19 answers were collected ( Coujard, 2022): this number cannot be considered as representative of the overall task force members, but it still gives some hints on how the approach was considered by the most active (and reactive) members. All respondents considered that the frequency of meetings was adequate ( *i.e.* “just the right number of meetings”). 18 respondents assessed the duration of meetings (1h30) as “just the right duration”, and one person considered it too short.

**Figure 3.**
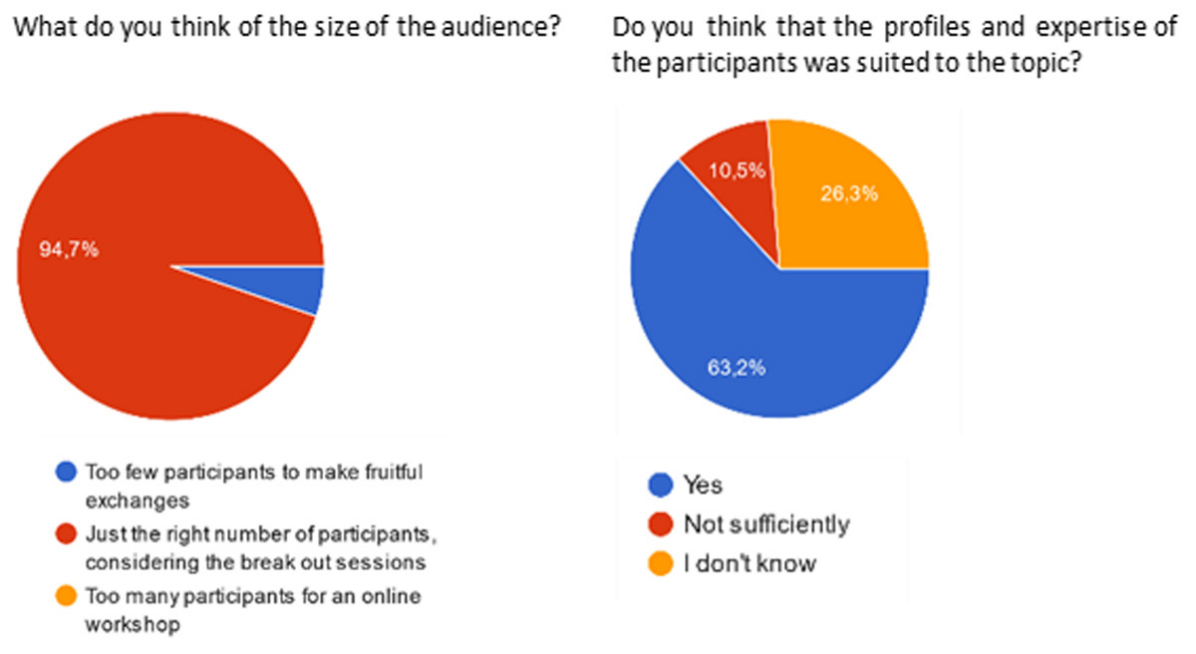
Participant feedback on the size and profile of the workshop’s audience.

**Figure 4.**
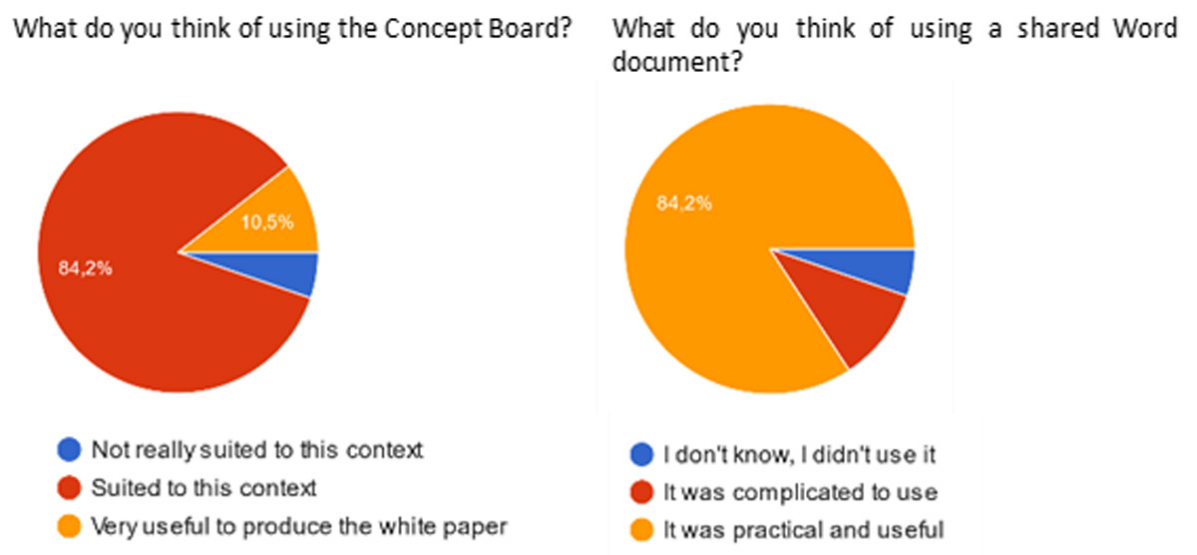
Participants’ feedback on the use of the concept board and share point.

Figure 3 and Figure 4 show the answers regarding the size and profile of the workshop audience, and the collaborative tools used. The audience size was deemed adequate by almost all participants. A majority of respondents estimated that the audience profile and expertise was suited to the topic investigated.

Regarding the tools used, a large majority of answers approved the use of the ConceptBoard and Sharepoint software used to brainstorm online and share the draft white paper document.

The final and open question in the questionnaire was formulated as follows: “How could we improve the recruitment/selection of participants, the interactivity of sessions, and the written contributions of participants?”. Most answers dealt with the recruitment of participants and suggested to increase the communication efforts using social media, emailing, European clusters and platforms, and a project of publication as a motivational driver. Regarding the selection of experts, one respondent suggested to first draft the key ideas of each topic, as a driver to select adequate experts. We shall however recall that experts joined on a volunteer basis, according to their interest and knowledge on the topic, and willingness to invest a bit of time in this collaborative adventure – therefore there was no ‘selection’ *per se*. However, specifying each topic more precisely at the beginning of the process could indeed help experts better assess whether their expertise matches the topic.

## Conclusions

The collaborative process developed was tested over 18 months and implemented on 12 different topics relying on 27 brainstorming workshops. Building on the collective knowledge and experience of 135 participants and at least 31 EU-funded projects, it enabled to identify a significant series of research & innovation gaps related to smart buildings, that will next feed a strategic research and innovation agenda.

One limitation can be observed in the process: as it relied on volunteering, there was no selection or evaluation of the participants’ expertise to contribute. However, the collective sessions showed overall some clear convergence on the gaps identified. The feedback from the open consultation phase, though limited, led to some additions in research and innovation gaps but did not question the ones proposed by the task force members. It can therefore be carefully concluded that the gaps identified reach a consensus among the targeted innovation community.

The feedback collected on the process itself shows that the frequency, duration and attendance of the brainstorming workshops proposed were very relevant, while the selection of online participatory tools could still be improved.

This process could be replicated in other frameworks where research and innovation gaps are sought for, such as EC-funded Coordination and Support Actions, as well as sectoral roadmaps.

## Ethics and consent

After completion of the Horizon Europe proposal ethics self-assessment, no ethics issues were identified, therefore ethics approval are not required. With regard to the anonymous online poll mentioned in section 3.3, consent for use of this data was implied by completion of the online survey.

## Data Availability

Zenodo: Feedback from task force members on the format, frequency and duration of workshop. https://doi.org/10.5281/zenodo.7418966 ( Coujard, 2022) This project contains the following underlying data: Feedback.xlsx Zenodo: Online live questionnaire to task force members on the workshop format and collaborative tools used. https://doi.org/10.5281/zenodo.7458116 ( Coujard, 2022b) This project contains the following extended data: Questionnaire.xlsx Data are available under the terms of the Creative Commons Attribution 4.0 International license (CC-BY 4.0). ^1^ See the twelve SmartBuilt4EU white papers that provide a state of the art: https://smartbuilt4eu.eu/publications/ ^2^ See for instance the synthetic diagram proposed in page https://www.cleantech.com/smart-buildings-and-space-utilization-value-chain/ ^3^ Details are provided on the related web pages of the SmartBuilt4EU website: https://smartbuilt4eu.eu/task-forces/ ^4^ The Expert Board gathered a dozen of experts from various fields connected to smart buildings, who committed to contribute the SmartBuilt4EU project, through commitment letters signed at project proposal stage. The Expert Board met online approximately every 6 months to discuss the project activities, including the chairing of the various task force topics. Co-chair are listed in the respective SmartBuilt4EU White Papers (see footnote 3) ^5^ DOWEL bought a license to this tool, enabling then any task force members to freely connect and use the tool, through a login as “invited member”
