# Supplementary material for: Collaborative design approach to identify research and innovation needs within the European smart building community
Source: Open Res Eur. 2024 Oct 15;3:36. Originally published 2023 Feb 20. [Version 2] doi: 10.12688/openreseurope.15174.2 (PMC11484537; doi:10.12688/openreseurope.15174.2)
Supplement: Peer review history

# Reviewer report: Reviewer response for version 2

**DOI:** 10.21956/openreseurope.18052.r45171

**Published:** 2024-10-16

**Authors:** Regis Decorme, Mojtaba Maktabifard

**Affiliations:** 1 Innovation, R2M Solution, Roquefort-les-Pins, Alpes Maritimes, France; 2 Innovation, R2M solution, Roquefort les Pins, France

All comments have been well integrated. Thank you very much.

Is the study design appropriate and does the work have academic merit?

Yes

Is the work clearly and accurately presented and does it cite the current literature?

Yes

If applicable, is the statistical analysis and its interpretation appropriate?

Yes

Are all the source data underlying the results available to ensure full reproducibility?

Yes

Are the conclusions drawn adequately supported by the results?

Yes

Are sufficient details of methods and analysis provided to allow replication by others?

Yes

Reviewer Expertise:

Environmental Engineering

We confirm that we have read this submission and believe that we have an appropriate level of expertise to confirm that it is of an acceptable scientific standard.

# Reviewer report: Reviewer response for version 1

**DOI:** 10.21956/openreseurope.16413.r30921

**Published:** 2023-03-03

**Authors:** Regis Decorme, Mojtaba Maktabifard

**Affiliations:** 1 Innovation, R2M solution, Roquefort les Pins, France; 2 Innovation, R2M Solution, Roquefort-les-Pins, Alpes Maritimes, France

The manuscript titled “Collaborative design approach to identify research and innovation needs within the European smart building community”, identifies research and innovation gaps related to smart buildings. The article deals with an increasingly important issue of enhancing sustainability of buildings through different criteria such as energy efficiency and optimization of resource management.

 The authors begin with emphasizing that, although several ongoing developments are dedicated to upgrading the intelligence of the buildings, however, the ongoing efforts are dispersed. This paper adds value to the literature by sharing an overview of what is achieved today in Europe and highlighting research and innovation gaps which should be dealt with in future. The authors applied a collaborative design approach, comprising gathering maximum stake-holders, 3 brainstorming workshops, drafting white papers and collecting feedbacks on them. 12 different topics were implemented in frame of workshops and 135 people participated who were mostly experienced with EU-funded projects. I would recommend the authors to enhance the quality of Figure 1 to better demonstrate the details of the applied methodology. Nevertheless, the paper well presents the statistical analysis derived from the feedbacks of participants. The work is an outcome of SmartBuilts4EU project and would be interesting for the addresees of Open Research Europe publications.

 Here are my minor editorial recommendations for the authors to increase the quality of the paper:

- Page 3: I would use the term “to change” instead of “to changing”.
- Page 5: Please enhance the quality of Figure 1.
- Page 5: Add the term stakeholders “who were” not directly involved.
- Page 5: Workshops were “more” promoted; change to workshops were “better” promoted.
- Page 5: “one workshop was organized within the Sustainable Places 2022 conference in September 2022.”

Could you provide any reference/link to the aforementioned conference?

- Page 5: Use “topic A” instead of plural form.
- Page 5: I would recommend using “featured” (past tense) in the sentence.

Is the study design appropriate and does the work have academic merit?

Yes

Is the work clearly and accurately presented and does it cite the current literature?

Yes

If applicable, is the statistical analysis and its interpretation appropriate?

Yes

Are all the source data underlying the results available to ensure full reproducibility?

Yes

Are the conclusions drawn adequately supported by the results?

Yes

Are sufficient details of methods and analysis provided to allow replication by others?

Yes

Reviewer Expertise:

Environmental Engineering

We confirm that we have read this submission and believe that we have an appropriate level of expertise to confirm that it is of an acceptable scientific standard, however we have significant reservations, as outlined above.

# Response

**Published:** 2023-10-05

**Authors:** Clémentine COUJARD

**Affiliations:** DOWEL Innovation, France

Dear reviewer, Thanks for your feedack! please find below my reponses:  

- Page 3: I would use the term “to change” instead of “to changing”: changing is used in this sentence as an adjective (i.e. changing or evolving conditions). Your proposal would change the sentence meaning, which I prefer to avoid.
- Page 5: Please enhance the quality of Figure 1. Unfortunately I cannot obtain any better definition of this image from the software.
- Page 5: Add the term stakeholders “who were” not directly involved. Ok taken into account
- Page 5: Workshops were “more” promoted; change to workshops were “better” promoted. Ok taken into account
- Page 5: “one workshop was organized within the Sustainable Places 2022 conference in September 2022.” Could you provide any reference/link to the aforementioned conference? As this article is published as part of this Conference proceedings, I assume that such references are already clearly established.

- Page 5: Use “topic A” instead of plural form. Sorry I do not understand this comment, can you please mention clearly the sentence to be addressed?

- Page 5: I would recommend using “featured” (past tense) in the sentence. Ok taken into account

# Reviewer report: Reviewer response for version 1

**DOI:** 10.21956/openreseurope.16413.r30924

**Published:** 2023-02-27

**Authors:** Abdul Samad Kazi

**Affiliations:** 1 VTT Technical Research Centre of Finland, Espoo, Finland

This is a very well-written article that uses a collaborative design approach (many interactive workshops) to identify current R&D gaps and innovation needs to support the acceleration of smart and energy-efficient buildings in Europe (in particular) from a user centric perspective.

 It clearly defines key barriers, challenges, and uses an interactive approach to overcome these. 

 The results and findings are based on 12 white papers prepared through the active participation and discussion of 135 participants across 23 European countries. Results and findings of 30+ relevant EU projects were also considered.

Is the study design appropriate and does the work have academic merit?

Yes

Is the work clearly and accurately presented and does it cite the current literature?

Yes

If applicable, is the statistical analysis and its interpretation appropriate?

Yes

Are all the source data underlying the results available to ensure full reproducibility?

Yes

Are the conclusions drawn adequately supported by the results?

Yes

Are sufficient details of methods and analysis provided to allow replication by others?

Yes

Reviewer Expertise:

smart cities, innovation ecosystems, construction ICT

I confirm that I have read this submission and believe that I have an appropriate level of expertise to confirm that it is of an acceptable scientific standard.
